# AQP0 is a novel surface marker for deciphering abnormal erythropoiesis

**DOI:** 10.1186/s13287-021-02343-4

**Published:** 2021-05-06

**Authors:** Tso-Fu Wang, Guan-Ling Lin, Sung-Chao Chu, Chang-Chin Chen, Yu-Shan Liou, Hsin-Hou Chang, Der-Shan Sun

**Affiliations:** 1 Departments of Hematology and Oncology, Hualien Tzu Chi Hospital, Buddhist Tzu Chi Medical Foundation, Hualien, Taiwan, Republic of China; 2 College of Medicine, Tzu-Chi University, Hualien, Taiwan, Republic of China; 3 Department of Molecular Biology and Human Genetics, Tzu-Chi University, No. 701, Section 3, Zhong-Yang Road, Hualien, 97004 Taiwan, Republic of China; 4 Department of Laboratory Medicine, Hualien Tzu Chi Hospital, Buddhist Tzu Chi Medical Foundation, Hualien, Taiwan, Republic of China

**Keywords:** Aquaporin 0 (AQP0), Erythropoiesis, Dyserythropoiesis, Hematopoiesis, Bone marrow, Flow cytometry, Biomarker

## Abstract

**Background:**

Hematopoiesis occurs in the bone marrow, producing a complete spectrum of blood cells to maintain homeostasis. In addition to light microscopy, chromosome analysis, and polymerase chain reaction, flow cytometry is a feasible and fast method for quantitatively analyzing hematological diseases. However, because sufficient specific cell markers are scarce, dyserythropoietic diseases are challenging to identify through flow cytometry.

**Methods:**

Bone marrow samples from C57BL/B6 mice and one healthy donor were analyzed using traditional two-marker (CD71 and glycophorin A) flow cytometry analysis. After cell sorting, the gene expressions of membrane proteins in early and late erythropoiesis precursors and in nonerythroid cells were characterized using microarray analysis.

**Results:**

Among characterized gene candidates, aquaporin 0 (AQP0) expressed as a surface protein in early- and late-stage erythropoiesis precursors and was not expressed on nonerythroid cells. With the help of AQP0 staining, we could define up to five stages of erythropoiesis in both mouse and human bone marrow using flow cytometry. In addition, because patients with dyserythropoiesis generally exhibited a reduced population of APQ0^high^ cells relative to healthy participants, the analysis results also suggested that the levels of APQ0^high^ cells in early erythropoiesis serve as a novel biomarker that distinguishes normal from dysregulated erythropoiesis.

**Conclusions:**

AQP0 was successfully demonstrated to be a marker of erythroid differentiation. The expression levels of AQP0 are downregulated in patients with dyserythropoiesis, indicating a critical role of AQP0 in erythropoiesis. Accordingly, the level of AQP0^high^ in early erythroid precursor cells may serve as a reference parameter for diagnosing diseases associated with dyserythropoiesis.

**Supplementary Information:**

The online version contains supplementary material available at 10.1186/s13287-021-02343-4.

## Background

Red blood cells (RBCs, also known as erythrocytes) constitute a major and critical cell type in the circulatory system; it is responsible for the delivery of oxygen to the entire body. Erythropoiesis is the process of erythrocyte production in adult bone marrow [1]. Impaired erythropoiesis leads to dyserythropoietic diseases, such as Diamond–Blackfan anemia, β-thalassemia, congenital dyserythropoietic anemia, myelodysplastic syndromes (MDS), and polycythaemia vera [2–6]. All types of blood cells are derived from hematopoietic stem cells (HSCs). To produce RBCs, HSCs differentiate into megakaryocyte-erythroid progenitors and then burst forming unit-erythroid (BFU-E) and colony-forming unit-erythroid (CFU-E). Upon erythropoietin (EPO) stimulation, CFU-E further develops into various erythroid precursors (proerythroblasts, basophilic erythroblasts, polychromatic erythroblasts, and orthochromatic erythroblasts). To form mature RBCs, the terminal maturation of erythroid cells involves two steps: (1) the condensation and expulsion of the nucleus to form reticulocytes and (2) the organelle clearance and remodeling of the membrane and proteome [1, 7].

Based on the differential expression levels of cell-surface molecules during erythropoiesis, flow cytometry analysis is a useful tool for analyzing the erythropoiesis process [8]. For example, the CD71-transferrin receptor and CD235a-glycophorin A (GPA; or TER119, a molecule associated with GPA) have been used to divide erythropoiesis into four distinct stages (proerythroblast, basophilic erythroblast, late basophilic erythroblast and polychromatic erythroblast, and orthochromatic erythroblast stages) [9–11]. In a recent study, GPA, band 3 (transmembrane protein of erythrocytes), and α4 integrin were used to distinguish human erythroid terminal differentiation into six distinct stages (proerythroblast, early basophilic, late basophilic, polychromatic, orthochromatic erythroblast, and reticulocytes stages) [12]. In addition to being applied to delineate normal erythropoiesis, flow cytometry analysis can also be clinically used for reticulocyte enumeration, fetomaternal hemorrhage detection, hemoglobin F quantitation in sickle cell disease, and hereditary spherocytosis evaluation when combined with specific antibodies or fluorescent reagents [13–17]. MDSs are hematological neoplasms with clonal alterations of those HSCs that are characterized by ineffective hematopoiesis and peripheral cytopenia [18–20]. MDS occurs in individuals older than 60 years and has recently become more common [21, 22]. Although cytomorphology and cytogenetics are the primary methods for diagnosis, flow cytometry is also a valuable tool to improve the diagnosis and classification of MDS [23, 24]. Approximately, 85% of MDS patients have dysplastic alterations in their erythroid lineage [25, 26]; however, compared with granulocytes, monocytes, and lymphocytes, the erythroid lineage requires more specific markers in flow cytometry [23].

In the present study, we discovered that aquaporin 0 (AQP0, which belongs to a family of water channels [27]) is a novel surface marker for identifying erythroid precursor cells. Although the biological function of AQP0 in erythropoiesis requires further characterization, the potential applications of using AQP0 to help delineate human erythropoiesis and diagnose dyserythropoietic diseases are likely to be feasible, as discussed in this paper.

## Methods

### Microarray analysis of human erythropoiesis stages

One residual specimen from a clinical bone marrow examination of a patient without anemia symptoms was collected from Buddhist Tzu-Chi General Hospital (Hualien, Taiwan). The sample was centrifuged at 300 *g* for 5 min at room temperature. The buffy coat fraction was collected and diluted with phosphate-buffered saline (PBS) at a 1:1 ratio before being loaded on Ficoll-Paque PLUS (GE Healthcare, Uppsala, Sweden) at a 4:3 ratio. The bottom fraction (containing matured erythrocytes) was depleted after a 400 *g* density centrifugation for 30 min at room temperature. The residual fraction was centrifuged at 300 *g* for 5 min at room temperature, and the cell fraction was collected and stained with anti-CD45 antibodies that were conjugated with peridinin chlorophyll protein complex (PerCP; BD Biosciences), anti-CD71 antibodies conjugated with phycoerythrin (PE; BD Biosciences), and anti-CD235a antibodies conjugated with fluorescein isothiocyanate (FITC; eBioscience); the cell fraction was then analyzed using flow cytometry (FACSAria II, BD). A portion of CD45^high^ cells was sorted as nonerythroid cells. CD45^−/dim^/SSC^low^ cells were gated and analyzed further. CD71^high^/CD235a^low^ and CD71^high^/CD235a^high^ were defined as the early and late stages of erythropoiesis, respectively. Sorted cells (nonerythroid and early and late stages of erythropoiesis) were analyzed for global gene expression using a Phalanx Human OneArray® Gene Expression HOA 6.1 (Phalanx Biotech Group, Taiwan) microarray.

### Microarray analysis of mouse erythropoiesis stages

Bone marrow cells were isolated from C57BL/6J mice (male, 11 weeks old) by flushing their femurs and tibiae with serum-free RPMI medium (Biowest) containing anticoagulant acid-citrate-dextrose (ACD) at a 9:1 ratio. Cells were collected after centrifugation at 300 *g* for 5 min at room temperature and stained with anti-CD71 antibodies conjugated with FITC (BioLegend) and with anti-TER119 antibodies conjugated with allophycocyanin (APC; BD Biosciences). Flow cytometer was used to analyze the cell populations (FACSAria II, BD). A portion of CD71^−/dim^/TER119^−/dim^ cells was sorted as nonerythroid cells. CD71^high^/TER119^low^ and CD71^high^/TER119^high^ cells were sorted into the early stage and late stage of erythropoiesis, respectively. We used a Phalanx Mouse OneArray Gene Expression MOA2.1 (Phalanx Biotech Group, Taiwan) microarray to study the global gene expression of the three sorted cell populations (i.e., nonerythroid stage and early and late stages of erythropoiesis).

### Flow cytometry analysis

#### Human bone marrow

Residual specimens from a clinical bone marrow examination of healthy stem cell donors or patients were collected from Buddhist Tzu-Chi General Hospital (Hualien, Taiwan). Matured erythrocytes were lysed after the addition of 50 mL of ammonium-chloride-potassium buffer (0.15 M NH_4_Cl, 10 mM KHCO_3_, 0.1 mM Na_2_EDTA, pH 7.2–7.4) for 10 min at room temperature. Cells were washed with PBS containing 2 mM ethylenediaminetetraacetic acid (EDTA) and 0.5% bovine serum albumin (BSA) and centrifuged at 300 *g* for 5 min at room temperature. The cell pellets were dissolved in 500 μL PBS containing 2 mM EDTA and 0.5% BSA before being loaded on 2 mL 100% fetal bovine serum after filtration with a 55-μm mesh. Ghost erythrocytes were depleted after the supernatant was removed after centrifugation at 300 *g* for 5 min at room temperature. The residual cells were stained with anti-CD71 antibodies conjugated with PE/Cy7 (BioLegend), anti-CD235a antibodies conjugated with FITC, and anti-AQP0 antibodies conjugated with Alexa Fluor 647 (AF647; Bioss) for 30 min at 4°C. After being washed with PBS, cells were resuspended in 500 μL of PBS and analyzed using flow cytometry (FACSCalibur, BD) and Kaluza analysis software (Beckman Coulter).

#### Mouse bone marrow

Bone marrow cells were isolated from C57BL/6J mice (male, 15–29 weeks old) by flushing femurs and tibiae with PBS containing 2 mM EDTA and 0.5% BSA. Cells were collected after being passed through 55-μm mesh and incubated with PBS containing 2 mM EDTA and 5% BSA to block any nonspecific binding. Cells were then stained with anti-CD71 antibodies conjugated with FITC, anti-TER119 antibodies conjugated with PE/Cy7 (BioLegend), and anti-Aqp0 antibodies conjugated with AF647 (Bioss) for 1 h at room temperature. After being washed with PBS, cells were resuspended in 1 mL of PBS and analyzed using flow cytometry (FACSAria II, BD) and Kaluza analysis software (Beckman Coulter). Nonerythroid, R1, R2, R3, and R4 cell populations were sorted using flow cytometry (FACSAria II, BD).

### Quantitative reverse transcription polymerase chain reaction

Total RNA was isolated from sorted cells using TRIzol reagent (Invitrogen) per the manufacturer’s instructions. Genomic DNA was eliminated using DNase (Promega). The purity and concentration of RNA was quantified using a Nanodrop spectrophotometer (ThermoFisher Scientific). The reverse transcription of RNA (1 μg/sample) was performed using an iScript cNDA Synthesis kit (Bio-Rad) per the manufacturer’s instructions. Quantitative reverse transcription polymerase chain reaction (qRT-PCR) was conducted using SYBR Green Real-Time PCR Master Mixes (ThermoFisher Scientific) and a StepOnePlus Real-Time PCR System (Applied Biosystems). The results were normalized with glyceraldehyde 3-phosphate dehydrogenase (GAPDH) and fold changes were calculated with the formula 2^−ΔΔCT^. The primer sequences used for qRT-PCR are listed as follows (from the 5′ end to 3′ end): Klf1 (forward-TACACCAAGAGCTCGCACC; reverse-TGGTCAGAGCGTGAAAAAGC), Nfe2 (forward-CTCCTCAGCAGAACAGGAACA; reverse-GCAATATGTTGGAGGTGGCAG), Gfi1b (forward-GTTGCTGAACCAGAGCCTTC; reverse-GGGGGTCTGTGTGTAGCTGT), and GAPDH (forward-TCAACAGCAACTCCCACTCTTCCA; reverse-ACCCTGTTGCTGTAGCCGTATTCA).

### Statistics

All data are presented in terms of their mean ± standard deviation and analyzed in Microsoft Excel 2010. A two-tailed Student *t* test was conducted to compare between groups. Statistical significance was indicated if *P* < 0.05.

## Results

### *AQP0* was selected as a gene that encoded surface proteins specifically expressed in the early and late erythropoiesis stages using microarray analysis

**Fig. 1:**
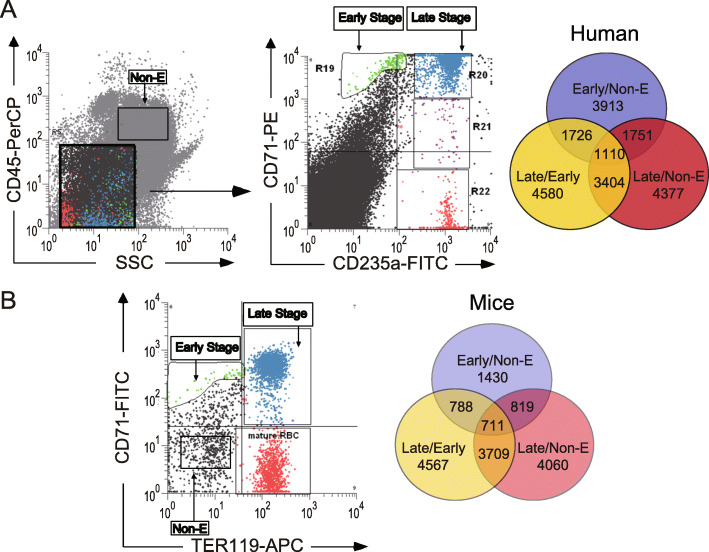
Microarray analysis of sorted erythroid differentiation stages for human and mouse bone marrow. Microarray analysis of sorted erythroid differentiation stages from human bone marrow (**a**). Human bone marrow cells were isolated and stained with anti-CD45 antibodies conjugated with PerCP, anti-CD71 antibodies conjugated with PE, and anti-CD235a antibodies conjugated with FITC. A portion of CD45^high^ cells was sorted as nonerythroid cells. CD45^−/dim^/SSC^low^ cells were gated and analyzed further. CD71^high^/CD235a^low^ and CD71^high^/CD235a^high^ were defined as indicating the early stage and late stage of erythropoiesis, respectively. The numbers 3913 and 4377 indicate the genes that are specifically expressed in the early and late erythroid differentiation stages relative to nonerythroid cells. The number 4580 indicates the gene that is specifically expressed in the late erythroid differentiation stage relative to the early erythroid differentiation stage. The numbers 1751, 3404, and 1726 indicate the gene shared with each pair (early/nonerythroid vs. late/nonerythroid, late/nonerythroid vs. late/early, and late/early vs. early/nonerythroid). The number 1110 indicates the gene shared with all three groups (early/nonerythroid, late/nonerythroid, and late/early). Microarray analysis of sorted erythroid differentiation stages from mouse bone marrow (**b**). Mouse bone marrow cells were isolated and stained with anti-CD71 antibodies conjugated with FITC and anti-TER119 antibodies conjugated with APC. A portion of CD71^−/dim^/TER119^−/dim^ cells was sorted as nonerythroid cells. CD71^high^/TER119^low^ and CD71^high^/TER119^high^ were defined as indicating the early stage and late stage of erythropoiesis, respectively. The numbers 1430 and 4060 indicate the genes that are specifically expressed in early and late erythroid differentiation stages relative to nonerythroid cells. The number 4567 indicates the gene that is specifically expressed in the late erythroid differentiation stage relative to the early erythroid differentiation stage. The numbers 819, 3709, and 788 indicate the genes shared with each pair (early/nonerythroid vs. late/nonerythroid, late/nonerythroid vs. late/early, and late/early vs. early/nonerythroid). The number 711 indicates the gene that is shared by all three groups (early/nonerythroid, late/nonerythroid, and late/early)

To identify genes specifically expressed in the early and late erythropoiesis stages, bone marrow cells from one patient without anemia symptoms were isolated and stained with antibodies against CD45, CD71, and CD235a. A portion of CD45^high^ cells was sorted as nonerythroid cells. In the flow cytometry, cell granularity was indicated by side scatter (SSC). CD45^−/dim^/SSC^low^ cells were gated and analyzed further. CD71^high^/CD235a^low^ and CD71^high^/CD235a^high^ were defined as indicating the early stage and late stage of erythropoiesis, respectively (Fig. 1a). To compare the erythropoiesis between humans and mice, bone marrow cells from 20 C57BL/6J mice were also isolated and stained with antibodies against CD71 and TER119. A portion of CD71^−/dim^/TER119^−/dim^ cells was sorted as nonerythroid cells. CD71^high^/TER119^low^ and CD71^high^/TER119^high^ were defined as indicating the early stage and late stage of erythropoiesis, respectively (Fig. 1b). The number of genes specifically expressed in the early and late erythropoiesis stages relative to nonerythroid cells in humans was 1751 according to the microarray analysis (log2 ratio ≥ 1; Fig. 1a), and 63 genes encoding cell membrane proteins among them were selected as candidate erythroid surface markers using the cellular component of the method of gene set enrichment analysis. To remove some low-expressed genes, we used the intensity of CD235a in the early erythropoiesis stage as a threshold (because CD235a is supposedly negative in the early erythropoiesis stage). In addition, to narrow down the set of genes that are specifically expressed in the early and late erythropoiesis stages to a manageable number, antibody availability and expression status (coexpressed in humans and mice) were used as prerequisites, with *AQP0* being one of these genes.

### Erythropoiesis could be distinguished into five stages (R1, R2, R3/Aqp0^−^, R3/Aqp0^+^, and R4) using three surface markers (CD71, TER119, and Aqp0) in mice

**Fig. 2:**
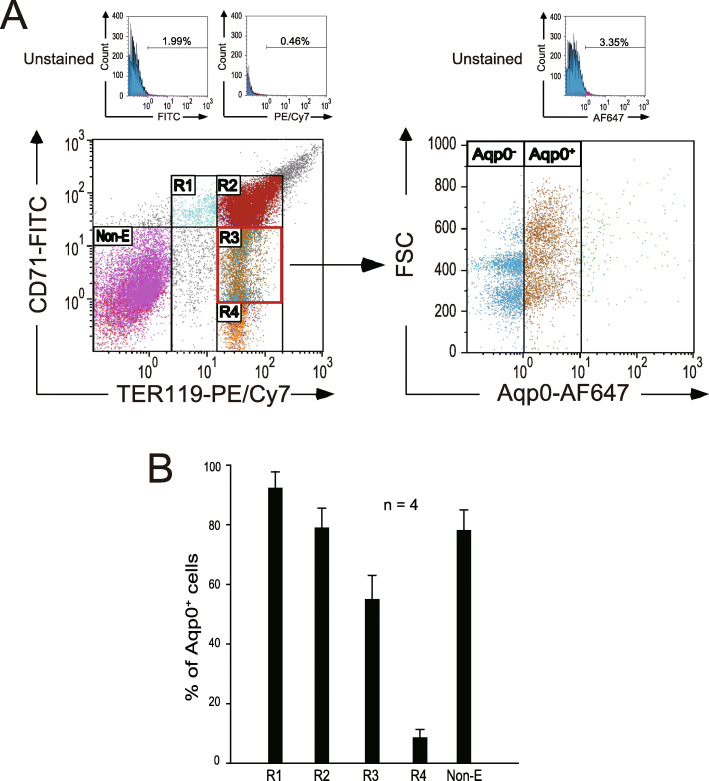
Characterization of mouse erythropoiesis stages using three surface markers (CD71, TER119, and Aqp0). Flow cytometry analysis of mouse erythropoiesis (**a**). Mouse bone marrow cells were isolated and stained with anti-CD71 antibodies conjugated with FITC, anti-TER119 antibodies conjugated with PE/Cy7, and anti-Aqp0 antibodies conjugated with Alexa Fluor 647. The percentages of positive cells of each antibody in unstained control were showed on the top. CD71^−/dim^/TER119^−/dim^ cells were gated as nonerythroid cells. CD71^high^/TER119^low^, CD71^high^/TER119^high^, CD71^dim^/TER119^high^, and CD71^−^/TER119^high^ were defined as region 1 (R1), region 2 (R2), region 3 (R3), and region 4 (R4), respectively. R3 was gated, and the relationship between FSC (cell size) and Aqp0 expression was analyzed. The percentages of Aqp0^+^ cells in each population were analyzed and quantified (**b**). Data are representative of four independent experiments and reported as the mean ± standard deviation

**Fig. 3:**
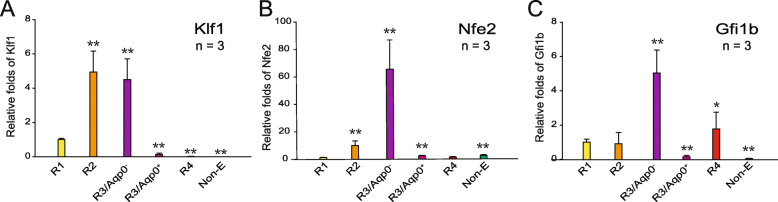
Relative mRNA expressions of erythroid specific transcription factors in each erythroid differentiation stage. Mouse bone marrow cells were isolated and categorized as R1, R2, R3/Aqp0^−^, R3/Aqp0^+^, R4, and nonerythroid populations on the basis of the fluorescence intensity of three erythroid specific cell surface markers (CD71, TER119, and Aqp0). The mRNA expression level of each gene in the R1 region was normalized to one-fold. Relative folds of mRNA expression of Klf1 (**a**), Nfe2 (**b**), and Gfi1b (**c**) in each cell population relative to R1 were analyzed using a quantitative reverse polymerase chain reaction assay. Data are representative of three independent experiments and reported as the mean ± standard deviation. *P < 0.05 and **P < 0.01 relative to R1 groups

To investigate whether *AQP0*-encoded proteins can be used as a surface marker to delineate erythropoiesis in humans, mouse bone marrow cells were first used to adjust the condition for flow cytometry. Flow cytometry analysis was performed after antibodies were used against CD71, TER119, and Aqp0 to stain the bone marrow cells. CD71^−/dim^/TER119^−/dim^ cells were gated as nonerythroid cells. On the basis of the expression levels of two known erythroid surface markers (CD71 and TER119), erythropoiesis was divided into the following four sequential stages: CD71^high^/TER119^low^ (region 1, R1), CD71^high^/TER119^high^ (region 2, R2), CD71^dim^/TER119^high^ (region 3, R3), to CD71^−^/TER119^high^ (region 4, R4; Fig. 2a) per previous studies [9–11, 28]. Our data revealed that the percentages of Aqp0^+^ cells in R1, R2, R3, and R4 were 92.9%, 78.8%, 54.8%, and 8.5%, respectively (Fig. 2b). Facilitated by Aqp0 staining, R3 could be further divided into two populations [R3/Aqp0^−^ (blue dots) and R3/Aqp0^+^ (brown dots)], as illustrated in Fig. 2a. The cells of these two populations, randomly distributed in R3, could not be easily separated by two erythroid surface markers (CD71 and TER119; Fig. 2a) or cell size (forward scatter, FSC; Fig. 2a). To determine differentially expressed genes of these two populations, a qRT-PCR assay was performed for sorted cell populations, including nonerythroid, R1, R2, R3/Aqp0^−^, R3/Aqp0^+^, and R4 populations. The data indicated that the mRNA expression levels of three well-known transcription factors for erythropoiesis (Klf1, Nfe2, and Gfi1b) [29–32] differed between these two cell populations (R3/Aqp0^−^ and R3/Aqp0^+^; Fig. 3a–c). These results suggested that Aqp0 is a novel and a feasible surface marker for analyzing erythropoiesis. With Aqp0, CD71, and TER119 staining, we could distinguish erythropoiesis into five newly identified differentiation stages.

### Percentages of AQP0^high^ cells in early erythroid cells served as a reference point to distinguish normal from dysregulated erythropoiesis

**Fig. 4:**
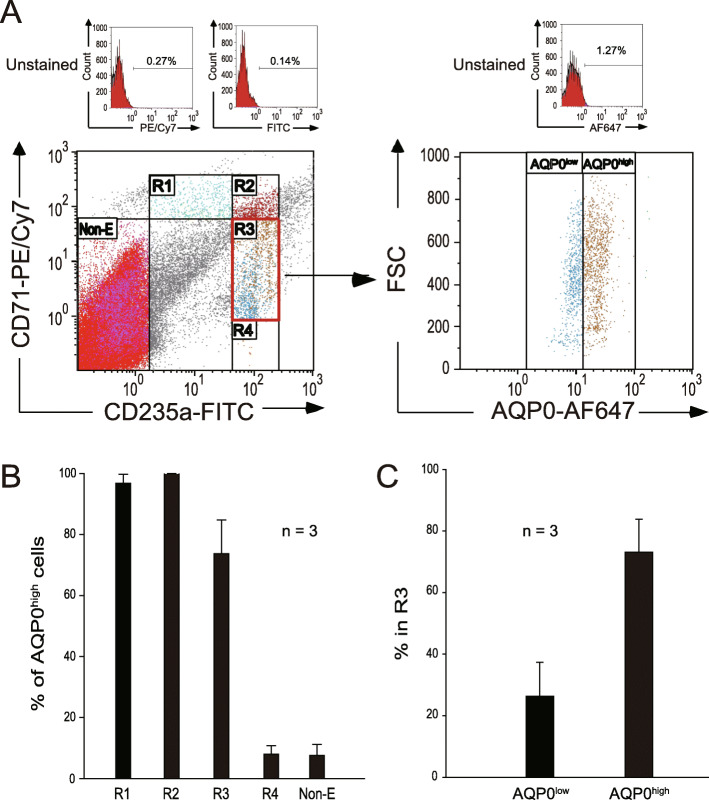
Characterization of human erythropoiesis stages using three surface markers (CD71, CD235a, and AQP0). Flow cytometry analysis of human erythropoiesis (**a**). Human bone marrow cells were isolated and stained with anti-CD71 antibodies conjugated with PE/Cy7, anti-CD235a antibodies conjugated with FITC, and anti-AQP0 antibodies conjugated with Alexa Fluor 647. The percentages of positive cells of each antibody in unstained control were showed on the top. CD71^−/dim^/CD235a^−/dim^ cells were gated as nonerythroid cells. CD71^high^/CD235a^low^, CD71^high^/CD235a^high^, CD71^dim^/CD235a^high^, and CD71^−^/CD235a^high^ were defined as region 1 (R1), region 2 (R2), region 3 (R3), and region 4 (R4), respectively. R3 was gated, and the relationship between FSC (cell size) and AQP0 expression was analyzed. The percentages of AQP0^high^ cells in each population were analyzed and quantified (**b**). The percentages of AQP0^low^ and AQP0^high^ cells in R3 were shown (**c**). The data are representative of three independent experiments and reported as the mean ± standard deviation

To evaluate the aforementioned three-marker staining in human erythropoiesis, bone marrow cells from three healthy volunteer donors were collected. Because high levels of mature RBCs may interfere with the staining, after a hypotonic depletion of these cells was conducted, bone marrow cells were labeled with CD71, CD235a, and AQP0 and then analyzed using flow cytometry. The CD71^−/dim^/CD235a^−/dim^ cells were gated as nonerythroid cells. CD71^high^/CD235a^low^, CD71^high^/CD235a^high^, CD71^dim^/CD235a^high^, and CD71^−^/CD235a^high^ were defined as R1, R2, R3, and R4, respectively (Fig. 4a). The intensity of AQP0 in humans was higher than in mice; thus, we calculated the percentage of AQP0^high^ in all cell populations (R1 96.8%, R2 99.9%, R3 73.7%, R4 8.0%, and nonerythroid 7.6%; Fig. 4b). As is the case in mice, R3 could also be distinguished into two populations (R3/AQP0^low^ and R3/AQP0^high^; Fig. 4c). These results collectively suggest that AQP0 is a novel and feasible surface marker for identifying erythropoiesis stages in both humans and mice.

**Fig. 5:**
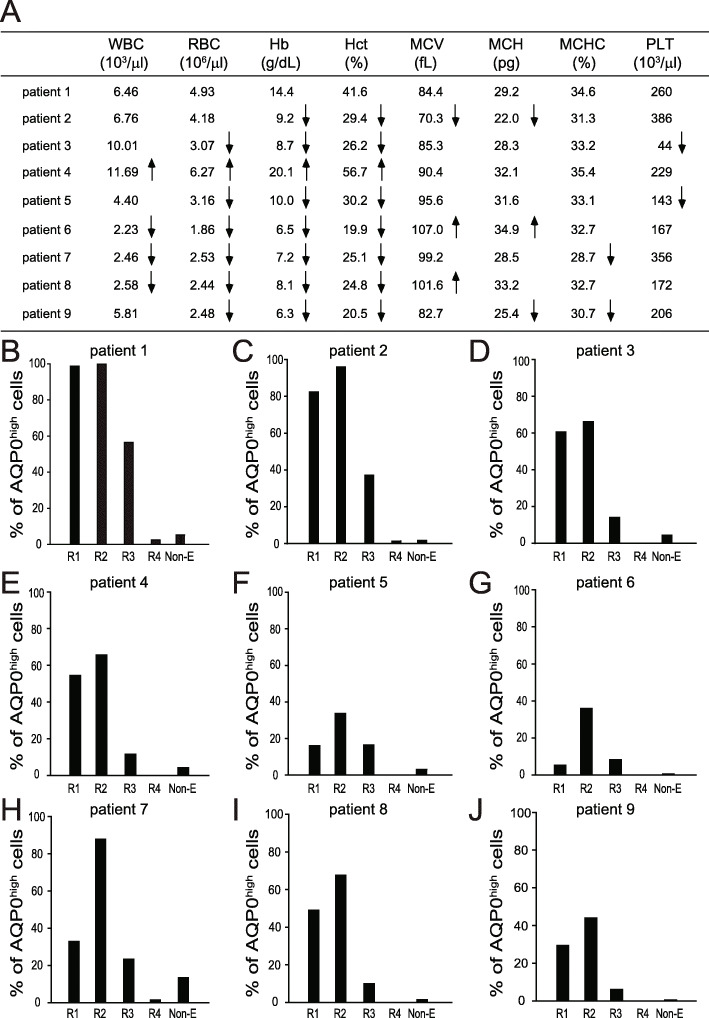
Characterization of erythropoiesis of patients using three surface markers (CD71, CD235a, and AQP0). Clinical complete blood count values of nine patients (**a**). The bone marrow cells from nine patients were isolated and stained with anti-CD71 antibodies conjugated with PE/Cy7, anti-CD235a antibodies conjugated with FITC, and anti-AQP0 antibodies conjugated with Alexa Fluor 647. CD71^−/dim^/CD235a^−/dim^ cells were gated as nonerythroid cells. CD71^high^/CD235a^low^, CD71^high^/CD235a^high^, CD71^dim^/CD235a^high^, and CD71^−^/CD235a^high^ were defined as region 1 (R1), region 2 (R2), region 3 (R3), and region 4 (R4), respectively. R3 was gated, and the relationship between FSC (cell size) and AQP0 expression was analyzed. The percentages of AQP0^high^ cells in each population were analyzed and quantified in patient 1 (**b**), patient 2 (**c**), patient 3 (**d**), patient 4 (**e**), patient 5 (**f**), patient 6 (**g**), patient 7 (**h**), patient 8 (**i**), and patient 9 (**j**). The data are representative of one independent experiment

To evaluate the potential of using AQP0 as a surface marker to aid the diagnosis of dyserythropoiesis, bone marrow cells from nine patients were collected and stained with antibodies against CD71, CD235a, and AQP0. Our data showed that the percentages of AQP0^high^ cells of patients in R1, R2, and R3 were both lower than those of healthy stem cell donors, except for patient 1 (Fig. 5b–j and Suppl. Figure 1 vs. Fig. 4a, b). These data were correlated with their clinical complete blood count (Fig. 5a). In patient 1, the percentages of AQP0^high^ cells in R1 and R2 were equal to that in healthy stem cell donors, and the percentage of AQP0^high^ cells in R3 was slightly less than that in healthy stem cell donors (Fig. 5b vs. Fig. 4b). Patient 1 had no abnormal clinical values pertaining to dyserythropoiesis (Fig. 5a). By contrast, the hemoglobin (Hb) and hematocrit (Hct) values of patients 2 and 7 (female) and patients 3, 5, 6, 8, and 9 (male) were both lower than normal (Hb for women 12.0–16.0 g/dL; Hb for men 13.5–17.5 g/dL; Hct for women 36.0–46.0%; Hct for men 41.0–53.0%). Moreover, mean corpuscular volume (MCV) in patient 2 was lower than normal (MCV: 80–100 fL), mean corpuscular hemoglobin (MCH) values in patients 2 and 9 were lower than normal (MCH: 26–34 pg), and RBC counts in patients 3, 5, 6, 7, 8, and 9 were lower than normal (RBC count for men 4.50–5.90 × 10^6^/μL; RBC count for women 4.00–5.20 × 10^6^/μL). The RBC count, Hb, and Hct value of patient 4 (male) were all higher than normal (Fig. 5a). These data indicated that the percentages of AQP0^high^ cells in early erythroid cells (especially R1 and R2) may serve as standards that aid in the diagnosis of dyserythropoiesis in humans.

## Discussion

This study demonstrated that AQP0 is expressed on early erythroid cells and that the percentages of AQP0^high^ in early erythroid cells (especially in R1 and R2) may serve as a standard that aids the diagnosis of dyserythropoiesis.

Aquaporins (AQPs) are a family of water channels that are responsible for the regulation of water flow inside and outside the cells. Fifteen mammalian AQPs have been identified thus far [27]. All AQPs organize as tetrameric pores in the cell membranes and each subunit contains six transmembrane spanning domains (both the N and C terminals are in the cytoplasm) with a molecular weight of approximately 30 kDa [33, 34]. In addition to water transport, some AQPs can also transport other molecules (glycerol, urea, or gases), maintain cellular structure, enable fluid flow across barrier tissues, and support cellular migration [33, 35]. The first identified water channel was originally named CHIP28 (now known as AQP1) and was discovered from erythrocyte plasma membrane by the Novel laureate Agre [36]. In addition to being expressed on early erythroid cells and mature erythrocytes [36–38], AQP1 is also expressed on the proximal convoluted tubules; on the descending thin limbs of the kidney; and in other secretory tissues, such as the choroid plexus, corneal epithelium, and gallbladder cholangiocytes [39, 40]. Previous study has found that AQP1 partitions into exosomes during reticulocyte maturation [41]. AQP1 can also upregulate the EDAG gene (also known as Hemgn in mice) and then increase hemoglobin expression to regulate erythroid differentiation [42]. Decreased AQP1 expression on the erythrocyte membranes was observed in patients with hereditary spherocytosis and was correlated with the severity of the disease [43]. Prior to our finding that AQP0 is expressed on early erythroid cells, studies have reported that AQP0 is the most abundant protein that is only expressed in the fiber cells of the eye lens where it is required for water permeability, hydrogen peroxide transportation and the maintenance of lens biomechanics, and transparency [44–46]. Humans and mice develop congenital cataracts when AQP0 has a genetic defect [46]. Our results demonstrate that AQP0 is expressed on early erythroid cells and that the percentages of AQP0^high^ in early erythroid populations (especially R1 and R2) may serve as a standard that aids the diagnosis of dyserythropoiesis. In addition, future studies should investigate why the low percentages of AQP0^high^ in early erythroid populations interfere with erythroid parameters (RBC number, Hb, Hct, MCV, and MCH), whether the level of AQP0 expression in early erythroid populations is correlated with the severity of dyserythropoiesis, and what functions AQP0 plays in erythropoiesis.

Previous studies have divided human erythropoietic cell populations into four consecutive differentiation stages (R1, R2, R3, and R4) using two-marker (CD71 and CD235a) flow cytometry. With the addition of the novel marker (AQP0) in this study, we can distinguish human erythropoietic precursor cells into five populations (R1, R2, R3/AQP0^low^, R3/AQP0^high^, and R4). In addition, although the exact timeline of R3/AQP0^low^ and R3/AQP0^high^ populations in erythropoiesis remains unclear, we found that three erythroid-differentiation critical transcription factors (Klf1, Nfe2, and Gfi1b) are differentially expressed in these two populations. Our contributions elucidate the molecular mechanism that underlies erythropoiesis.

## Conclusions

We found that AQP0 is not only expressed in the fiber cells of the eye lens but also in early erythroid cells. The percentages of AQP0^high^ in early erythroid cells (especially R1 and R2) may serve as a biomarker that aids the diagnosis of dyserythropoietic diseases.

## Supplementary Information

**Additional file 1: Figure S1.** Characterization of erythropoiesis of patients using three surface markers (CD71, CD235a, and AQP0). Flow cytometry analysis of erythropoiesis of patient 1 (A), patient 2 (B), patient 3 (C), patient 4 (D), patient 5 (E), patient 6 (F), patient 7 (G), patient 8 (H), and patient 9 (I). Patient bone marrow cells were isolated and stained with anti-CD71 antibodies conjugated with PE/Cy7, anti-CD235a antibodies conjugated with FITC, and anti-AQP0 antibodies conjugated with Alexa Fluor 647. CD71−/dim/CD235a−/dim cells were gated as nonerythroid cells. CD71high/CD235alow, CD71high/CD235ahigh, CD71dim/CD235ahigh, and CD71−/CD235ahigh were defined as region 1 (R1), region 2 (R2), region 3 (R3), and region 4 (R4), respectively. R3 was gated, and the relationship between FSC (cell size) and AQP0 expression was analyzed.

## Data Availability

All data generated and/or analyzed during this study are available from the corresponding author upon reasonable request.

## Abbreviations

RBCs: Red blood cells
AQP0: Aquaporin 0
HSCs: Hematopoietic stem cells
GPA: Glycophorin A
MDS: Myelodysplastic syndromes
PBS: Phosphate-buffered saline
PerCP: Peridinin Chlorophyll Protein Complex
PE: Phycoerythrin
FITC: Fluorescein isothiocyanate
APC: Allophycocyanin
EDTA: Ethylenediaminetetraacetic acid
BSA: Bovine serum albumin
qRT-PCR: Quantitative reverse transcription polymerase chain reaction
GAPDH: Glyceraldehyde 3-phosphate dehydrogenase
SSC: Side scatter
FSC: Forward scatter
Hb: Hemoglobin
Hct: Hematocrit
MCV: Mean corpuscular volume
MCH: Mean corpuscular hemoglobin
