# Supplementary material for: AQP0 is a novel surface marker for deciphering abnormal erythropoiesis
Source: Stem Cell Res Ther. 2021 May 6;12:274. doi: 10.1186/s13287-021-02343-4 (PMC8101103; doi:10.1186/s13287-021-02343-4)
Supplement: Supplementary file 1 — Additional file 1: Figure S1. Characterization of erythropoiesis of patients using three surface markers (CD71, CD235a, and AQP0). Flow cytometry analysis of erythropoiesis of patient 1 (A), patient 2 (B), patient 3 (C), patient 4 (D), patient 5 (E), patient 6 (F), patient 7 (G), patient 8 (H), and patient 9 (I). Patient bone marrow cells were isolated and stained with anti-CD71 antibodies conjugated with PE/Cy7, anti-CD235a antibodies conjugated with FITC, and anti-AQP0 antibodies conjugated with Alexa Fluor 647. CD71−/dim/CD235a−/dim cells were gated as nonerythroid cells. CD71high/CD235alow, CD71high/CD235ahigh, CD71dim/CD235ahigh, and CD71−/CD235ahigh were defined as region 1 (R1), region 2 (R2), region 3 (R3), and region 4 (R4), respectively. R3 was gated, and the relationship between FSC (cell size) and AQP0 expression was analyzed. [file 13287_2021_2343_MOESM1_ESM.zip › Legend for Fig-S1.pdf]

Fig. S1 Characterization of erythropoiesis of patients using three surface markers (CD71, CD235a, and AQP0). Flow cytometry analysis of erythropoiesis of patient 1 (A), patient 2 (B), patient 3 (C), patient 4 (D), patient 5 (E), patient 6 (F), patient 7 (G), patient 8 (H), and patient 9 (I). Patient bone marrow cells were isolated and stained with anti-CD71 antibodies conjugated with PE/Cy7, anti-CD235a antibodies conjugated with FITC, and anti-AQP0 antibodies conjugated with Alexa Fluor 647. $CD71^{-/dim}/CD235a^{-/dim}$ cells were gated as nonerythroid cells. $CD71^{high}/CD235a^{low}$, $CD71^{high}/CD235a^{high}$, $CD71^{dim}/CD235a^{high}$, and $CD71^{-}/CD235a^{high}$ were defined as region 1 (R1), region 2 (R2), region 3 (R3), and region 4 (R4), respectively. R3 was gated, and the relationship between FSC (cell size) and AQP0 expression was analyzed.
